# Infarction in the pars libera of the column of fornix including pre (cholinergic)- and post (circuit of Papez fiber tracts)-commissural fibers causes “basal forebrain” amnesia

**DOI:** 10.1007/s00234-015-1504-x

**Published:** 2015-03-03

**Authors:** Shunji Mugikura, Shoki Takahashi

**Affiliations:** Department of Diagnostic Radiology, Graduate School of Medicine, Tohoku University, 1-1 Seiryo-machi, Aoba-ku, Sendai, Japan 980-8574

Dear Sir:

We read with great interest the article entitled “Subcallosal artery stroke: infarction of the fornix and the genu of the corpus callosum. The importance of the anterior communicating artery complex. Case series and review of the literature” by Meila et al. [1].

The authors reported five cases of amnesia due to infarction of the subcallosal artery, the largest unpaired perforator of the anterior communicating artery (ACoA). These included three cases following clipping of an unruptured ACoA aneurysm, one case following coiling of the same, and one case probably due to microangiopathy unrelated to aneurysmal surgery.

We completely agree with the authors’ conclusion that despite its small size, the subcallosal artery is an important artery to monitor (to preserve) during surgical or endovascular treatment of ACoA aneurysms. Indeed, we examined whether 3D MR imaging using multiplanar reconstruction (MPR) could detect and locate subcallosal artery infarctions in 10 consecutive patients with amnesia after surgical repair of ACoA aneurysm [2]. The subjects fulfilled the criteria for amnesia based on formal neuropsychological examinations conducted in the chronic phase (a median time of 3 months after their surgery), when their amnesia was manifested. We found that all 10 patients had infarcts in the territory of the subcallosal artery, mostly bilaterally (nine of 10 patients). Therefore, we are in a position to confirm that both our study and the study by Meila et al. demonstrated a clear causal relationship between injury of the unpaired subcallosal artery during surgical clipping or coiling and amnesia.

We also agree with the authors’ conclusion that the column of fornix (FxCo), which constitutes a portion of the Papez neural circuit, is most strongly implicated in postoperative amnesia. Indeed, we found that the FxCo was involved in all patients, mostly bilaterally (nine of 10 patients), which mirrors the statistic of four of five patients in Meila et al. Moreover, in two of 10 patients in our series, the infarct was limited to the FxCo and the adjoining anterior commissure, with no other basal forebrain lesions visible in 3D MR imaging. Indeed, we discussed the same idea as Meila et al. as the most likely pathomechanism for the postoperative amnesia.

On the other hand, the cholinergic nuclei of the basal forebrain such as paraterminal gyrus including the septum pellucidum and diagonal band of Broca have long been considered as a primary cause of postoperative amnesia [3]. Therefore, postoperative amnesia is often called “basal forebrain” amnesia [4, 5]. Meila et al. did not find infarcts in regions of the basal forebrain other than the FxCo and the genu of the corpus callosum and consequently did not refer to the possible involvement of the cholinergic system. However, we would like to emphasize the potential relationship between cholinergic damage and amnesia in his study, based on the following two reasons.

The first concerns the anatomical characteristics of the pars libera of the FxCo, as described in the literature. We note that in the study of Meila et al., most of the patients appeared to have infarction foci in the pars libera of the FxCo. This, in itself, may indicate an interruption of the cholinergic system. As we discussed, the interruption of the cholinergic system is indicated by damage to the pars libera of the FxCo. In addition to post-commissural fibers, the pars libera of the FxCo incorporates pre-commissural fibers that include the cholinergic fibers connecting the septal nuclei, located in both the paraterminal gyrus and its adjacent septum pellucidum, to the hippocampus [6]. Thus, we suspect an association between damage to the cholinergic fibers within the pars libera of FxCo and amnesia, which we believe has not been previously mentioned.

**Fig. 1:**
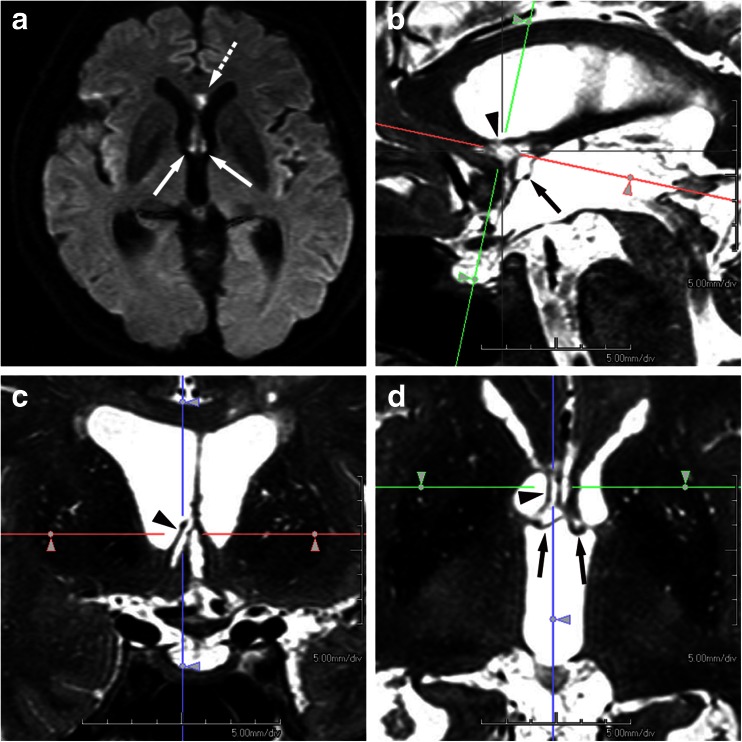
A 67-year-old man with a ruptured aneurysm of the ACoA was treated by transarterial coil embolization on the day of onset. Disorientation was suspected on the next day of embolization, but formal neuropsychological assessment was hardly valid due to confusional state in the acute phase. Diffusion-weighted image performed on the 4th day of embolization (**a**) demonstrates acute infarction in the column of fornix, bilaterally (*arrows*) and in the rostrum of the corpus callosum (*dashed arrow*). Two months later, formal neuropsychological tests revealed amnesia, confabulation, and disorientation. Paramedian sagittal (**b**), coronal (**c**), and axial (**d**) MPR images were generated from 3D data of T2-weighted volumetric isotropic turbo spin-echo acquisition (VISTA). The coronal image (**c**) corresponds to the *green line* in the paramedian sagittal (**b**), and the axial image (**d**) corresponds to the *red line* in the paramedian sagittal (**b**), so that both images pass through the superior part of the paraterminal gyrus in the right (*arrowheads* in **b**–**d**). Infarcted foci involve not only the pars libera of the column of the fornix bilaterally (*arrows* in **d**) and the rostrum of the corpus callosum (not shown) but also the paraterminal gyrus in the right and the anterior commissure (*arrow* in **b**). Thus, it may well be said that the involvement of the paraterminal gyrus and the anterior commissure found on 3D images in the chronic phase had not been visualized on the initial 2D diffusion-weighted images in the acute phase

The second reason for considering the involvement of the cholinergic system in such amnesia is that small subcallosal artery infarctions might have been missed by the relatively thick-slice 2D MR imaging technique used not only in the study of Meila et al. but also in other case reports. In fact, our study utilized 3D MR imaging using MPR and infarction included the paraterminal gyrus in eight patients and the diagonal band of Broca in five, which indicates the high prevalence of damage to the cholinergic nuclei. Although the arterial supply of the diagonal band of Broca has not been clearly defined, the subcallosal artery could supply eight regions including the preoptic area, paraterminal gyrus including a part of the septum pellucidum, subcallosal area, anterior commissure, and FxCo. Then, as previously described anatomically, it curves forward and upward to supply the rostrum, genu of the corpus callosum, and the anterior cingulate gyrus [7]. Therefore, we believe that an infarct in the subcallosal artery, frequently represented as a sagittally elongated vascular distribution along the characteristic S-shaped course of the artery in our study, could have involved the region of the cholinergic nuclei, including the paraterminal gyrus (Fig. 1). Thus, it is possible that small basal forebrain structures such as the cholinergic nuclei adjoining the FxCo might have been involved, but were missed or not clearly demonstrated, in the series that Meila et al. studied by 2D MR imaging.
